# Validation of NOViSE: A Novel Natural Orifice Virtual Surgery Simulator

**DOI:** 10.1177/1553350616669896

**Published:** 2016-09-26

**Authors:** Przemyslaw Korzeniowski, Daniel C. Brown, Mikael H. Sodergren, Alastair Barrow, Fernando Bello

**Affiliations:** 1 Imperial College London, London, UK

**Keywords:** NOTES, natural orifice surgery, flexible endoscopy, transgastric cholecystectomy, virtual reality simulator

## Abstract

The goal of this study was to establish face, content, and construct validity of NOViSE—the first force-feedback enabled virtual reality (VR) simulator for natural orifice transluminal endoscopic surgery (NOTES). Fourteen surgeons and surgical trainees performed 3 simulated hybrid transgastric cholecystectomies using a flexible endoscope on NOViSE. Four of them were classified as “NOTES experts” who had independently performed 10 or more simulated or human NOTES procedures. Seven participants were classified as “Novices” and 3 as “Gastroenterologists” with no or minimal NOTES experience. A standardized 5-point Likert-type scale questionnaire was administered to assess the face and content validity. NOViSE showed good overall face and content validity. In 14 out of 15 statements pertaining to face validity (graphical appearance, endoscope and tissue behavior, overall realism), ≥50% of responses were “agree” or “strongly agree.” In terms of content validity, 85.7% of participants agreed or strongly agreed that NOViSE is a useful training tool for NOTES and 71.4% that they would recommend it to others. Construct validity was established by comparing a number of performance metrics such as task completion times, path lengths, applied forces, and so on. NOViSE demonstrated early signs of construct validity. Experts were faster and used a shorter endoscopic path length than novices in all but one task. The results indicate that NOViSE authentically recreates a transgastric hybrid cholecystectomy and sets promising foundations for the further development of a VR training curriculum for NOTES without compromising patient safety or requiring expensive animal facilities.

## Introduction

Natural orifice transluminal endoscopic surgery (NOTES) is a surgical technique in which operations are performed by accessing a natural orifice such as the mouth, anus, urethra, or vagina, and passing through an internal incision to reach the operative site. It has the potential to bring about a paradigm shift in surgery by capitalizing on the benefits of established minimally invasive techniques; with a further reduction in postoperative pain, morbidity, and hospital stay.

There has been a marked increase in interest in NOTES since the first human cases in 2007.^1^ In response to this, leaders of The Society of American Gastrointestinal and Endoscopic Surgeons and the American Society for Gastrointestinal Endoscopy joined to form the Natural Orifice Surgery Consortium for Assessment and Research (NOSCAR). NOSCAR published a white paper outlining the potential benefits and risks associated with NOTES surgery.^2^ The paper highlighted that a key challenge in implementing NOTES into mainstream clinical practice is how to safely train operators in its application, particularly as it requires both endoscopic and surgical skills, hitherto chiefly acquired by gastroenterologists or surgeons in isolation. The need to avoid the high rate of surgical complications that occurred in the early days of laparoscopic surgery was also stressed.

Current enthusiasm for the potential benefits of NOTES, the lack of experienced NOTES surgeons, the growing body of evidence supporting VR simulation as an effective training modality, and proven interest in developing a NOTES VR simulator,^3^ led our team to develop a novel NOTES VR simulator: *NOViSE–Natural Orifice Virtual Surgery Simulator*.

The aim of this study is to establish the face, content, and construct validity for NOViSE when performing a transgastric hybrid NOTES cholecystectomy. This procedure was chosen as it remains the most commonly performed clinical application of natural orifice surgery.^4^ A transgastric approach was chosen to allow for the training of more advanced endoscopic skill set as, unlike transvaginal approaches in which the operator may pass the endoscope in a straight line from vagina to gallbladder, transgastric cholecystectomy involves near retroflexion of the endoscope. Moreover, the transgastric approach is more universal (ie, not limited to half of the population) and often more appealing to patients,^5^ with recent studies recommending further investigation to establish clear indications and guidelines for its use.^6,7^

## Materials and Methods

### Participants

#### Experts (Group A)

Experts were defined as surgeons who had performed 10 or more simulated (animal or ELITE simulator^8^) or human NOTES procedures independently. Our aim was to recruit between 5 and 10 experts. This is analogous to previous validation studies of a similar design.^9,10^

#### Novices (Group B)

Novices were defined as surgeons who had performed fewer than 10 animal or human NOTES procedures independently. In addition, in order to prevent construct validation of the simulator as a tool for acquiring basic endoscopic (as opposed to NOTES) skills, it was stipulated that novices must have performed at least 10 clinical endoscopic procedures independently. Equally, in order to prevent construct validation of the simulator as a tool for acquiring basic surgical skills, it was stipulated that novices must have performed at least 10 nonsimulated laparoscopic procedures independently. Our aim was to recruit between 5 and 10 novices as seen in previous studies.^9,10^

#### Gastroenterologists (Group C)

Gastroenterologists who had performed at least 1000 endoscopic procedures (as outlined above) independently on humans, but with no experience of laparoscopic procedures and with no or minimal NOTES experience were recruited to the study. The reason for separating Gastroenterologists from Novices is to compare which prior surgical experience, endoscopic or laparoscopic, has bigger impact on the acquisition of skills in NOTES.

#### Nonexperts (Group B + C)

Novices together with Gastroenterologists are referenced as Nonexperts further in the text.

### Participant Data

Participants’ operative experience, experience of videogames (shown to shorten time to proficiency in performing tasks on a validated laparoscopic VR simulator^11^) and demographic data were recorded using an online questionnaire through Survey Monkey.

### Simulator Description

**Figure 1.**
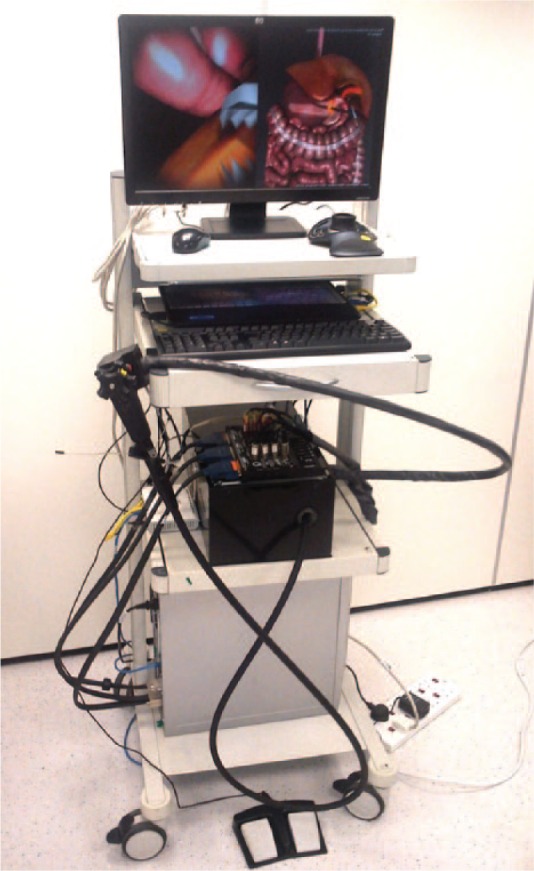
Simulator hardware setup.

NOViSE consists of a physical, force-feedback human-computer interface (the haptic device) and a real-time software simulation (the simulation). The haptic device comprises an enclosed black box of dimensions approximately 55 × 26 × 18 cm, into which passes a length of simulated endoscope shaft of 5 mm diameter through a small circular opening (Figure 1). The simulated endoscope shaft can be pushed or pulled through the opening (total travel 22 cm) and rotated freely. The angle and insertion distance are measured and read by the simulation software. DC motors connected to the simulated endoscope shaft inside the box provide both linear and rotational force feedback.

At the proximal end of the simulated endoscope shaft (approximately 1.5 m), a plastic replica of a standard endoscopic hand piece is attached. The hand piece consists of 2 thumb wheels, 2 push buttons, and 2 thin wires representing the endoscopic tool wires. Additionally, a double foot pedal is placed on the floor. This setup is similar to other validated endoscopy simulators.^12^ The amount of laparoscopic gallbladder retraction is controlled by pressing the keyboard keys (+/−).

The simulation software receives movements from the haptic device, calculates the motion of the virtual flexible endoscope, processes the interactions of the endoscope with internal organs, measures and stores the performance metrics, and calculates and sends the force feedback back to the haptic device.

### Simulator Tasks

To summarize, the simulation of a transgastric hybrid cholecystectomy involves navigating a flexible endoscope from a starting point in the esophagus, through a gastrostomy into the peritoneal cavity, identifying, clipping and ligating the cystic artery and duct, respectively, then dissecting the gallbladder off the liver bed with diathermy. The stages of the simulation are briefly outlined below.

**Figure 2.**
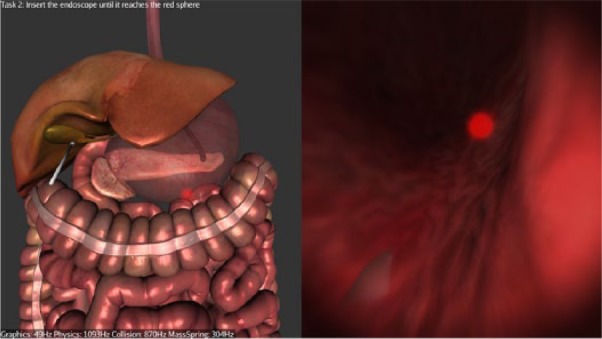
Navigating inside the stomach.

**Figure 3.**
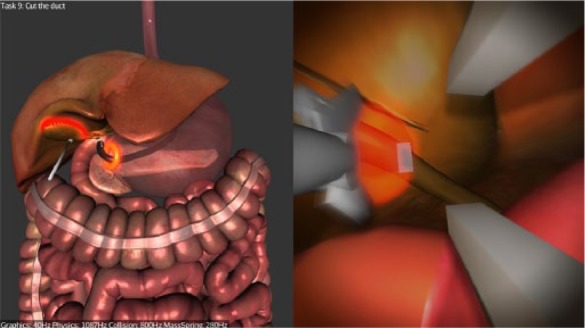
Cutting the cystic artery.

**Figure 4.**
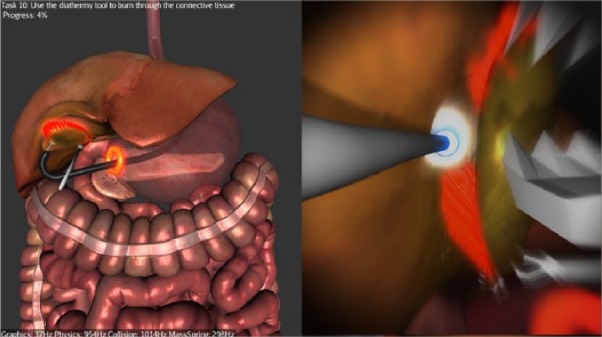
Dissecting the gallbladder off the liver bed with the diathermy.

*Stage 1*—*Navigation*: Participants are required to navigate to the first checkpoint (red glowing sphere) at the distal esophagus, from there to the second checkpoint (red glowing sphere) inside the stomach (Figure 2), and then from the stomach to the peritoneal cavity through the preexisting gastric incision.
*Stage 2—Clipping and Cutting*: Participants are required to clip the cystic artery at 2 prescribed points (indicated by glowing spheres), then to dissect it at a prescribed point (Figure 3). The same steps are then repeated for the cystic duct. The current version of the simulator does not replicate the dissection of Calot’s triangle.
*Stage 3—Diathermy*: Participants are required to use the diathermy tool to dissect the connective tissue attaching the gallbladder to the liver bed (Figure 4). Once this is completed, the simulation ends.

After each task is completed, the simulation automatically advances to the next task and appropriate instruments are automatically selected. The length of the simulator’s endoscope shaft is less than that of a real endoscope. If the available length is exceeded during insertion, the screen fades out, the simulation is paused and the user is asked to reset the insertion of the shaft by pulling its entire length out of the haptic device. This is a necessary trade-off to keep the simulator compact and portable.

### Simulation

All participants were required to complete 3 transgastric hybrid NOTES cholecystectomies in line with how clinical hybrid NOTES is currently being performed. Prior to performing their first procedure, all participants were given a technical instruction sheet outlining the nature of the simulation. The intention of this sheet was to give a brief overview of the equipment and tasks, as well as aspects of the simulation which were known to differ from reality owing to the limitations of performing the procedure on a simulator. Participants were also informed of what help they may receive from the researcher (who acted as assisting surgeon) during the procedure; namely, holding the endoscope in a particular position, advancing, retracting or activating the instruments and retracting the gallbladder. These actions would normally be performed by an assisting surgeon in real life. In order to prevent bias and control for any impact of the assistant’s role, the same researcher with experience in assisting during simulated surgeries was used throughout the study and was only permitted to act following a direct instruction from the participant. Additionally, the gallbladder retraction was restricted to being lifted or lowered always in the same direction through a keyboard combination (ie, the assistant could not freely manipulate the gallbladder). The instruction sheet was purposely designed to avoid instructing participants on the particular challenges of performing a NOTES cholecystectomy, so that any potential differences in performance between novices and experts could be better detected. After reading the instruction sheet, participants were given a maximum of 3 minutes to familiarize themselves with basic navigation of the endoscope and how to operate the instruments. This was done on a separate, nonanatomical simulated module utilizing simple geometrical shapes with no performance metrics recorded. Prior to commencing their first recorded procedure, participants were given the opportunity to ask questions relating to the practicalities of the simulation, but were not allowed to request any technical advice regarding how best to perform the procedure. No time limit was set for the 3 recorded procedures. Each procedure was performed at a time convenient to the participant. The data collection took place in the Surgical Innovation Centre at St Mary’s Hospital, Imperial College London, UK. Institutional review board approval was obtained from the Imperial College Research and Ethics Committee (Ref: ICREC_13_4_4).

### Metrics

The NOViSE software tracks and stores all the movements of the haptic device and of the virtual endoscope in real time. The software also stores a series of performance metrics during the simulation as shown in Table 1.

**Table 1.** Simulator Metrics.

For All Tasks	For Clipping and Cutting Tasks	For Gallbladder Dissection Tasks
• Task completion time Endoscope path length • Average and maximum force applied to tissue by tip of endoscope • Average and maximum force feedback	• Clipping/cutting distance from the indicated point (center of glowing sphere) • Clipping/cutting angle between the clipping/cutting tool and the surface of the duct (optimal = 90°) • Number of clippings/cuttings • Degree of instrument protrusion during the operation (one should avoid protruding the instruments from the tip of the endoscope when not in use to avoid unintentional damage to tissues)	• Number of diathermy activations • Total diathermy activation time • Time diathermy activated on non-target tissue • Time diathermy activated on target tissue • Percentage of diathermy activation on target tissue

### Validation

#### Face and Content Validity

Participants were asked to rate their agreement relating to face and content validity on a 5-point Likert-type scale (Strongly disagree; Disagree; Neutral; Agree; Strongly agree). Fifteen statements pertaining to the realism of the graphics, behavior of tools and tissues, overall difficulty, and overall realism of the simulation were used to assess face validity (Table 2). Six statements related to the adequacy of the simulated tasks and perceived utility of the simulator as a training tool for NOTES were used to assess content validity (Table 2). The statements were contained in the same questionnaire that assessed participant demographics and were phrased such that agreement correlated with face and content validity.

**Table 2.** Survey Questions.

Face validity questions:
• Q1: The endoscope clipper and scissors were visually realistic • Q2: The endoscope diathermy was visually realistic • Q3: The endoscope grasper was visually realistic • Q4: The tissues and organs were visually realistic • Q5: The endoscope hardware was visually realistic • Q6: The endoscope hardware felt realistic • Q7: The movement of the tip of the endoscope was realistic • Q8: The movement of the shaft of the endoscope was realistic • Q9: The amount and nature of ‘looping’ of the endoscope was realistic • Q10: The freedom of movement of the endoscope was realistic • Q11: The length of the endoscope was realistic • Q12: The interaction of the endoscope and instruments with the tissues was visually realistic • Q13: The interaction of the endoscope and instruments with the tissues felt realistic • Q14: The difficulty of the simulated procedure was realistic • Q15: Overall the simulator was realistic
Content validity questions:
• Q1: Navigation of the endoscope into the peritoneal cavity is a useful training tool for NOTES • Q2: Clipping and cutting the artery and the duct is a useful training tool for NOTES • Q3: Dissecting the gallbladder from the liver bed is a useful training tool for NOTES • Q4: The range of exercises provided by the simulator are sufficient to make it a useful training tool for NOTES • Q5: Overall the simulator is a useful training tool for NOTES • Q6: I would recommend the simulator to others

#### Construct Validity

Construct validity was evaluated by comparing operative performance metrics of experts, novices, and gastroenterologists.

### Data Analysis

All data were kept anonymous. Data were analyzed using the Statistical Package for the Social Sciences (SPSS 21). Descriptive statistics and frequencies were calculated with appropriate methods according to the type of data. Following evaluation, variables of nonparametric distribution were compared using the Mann-Whitney *U* test. Statistical significance was set at *P* < .05.

## Results

### Demographics and Procedural Experience

Demographic data of participants are shown in Table 3, while their procedural experience is shown in Table 4. Four experts were recruited to group A, 7 novices were recruited to group B, and 3 gastroenterologists were recruited to group C. Most experts and novices were upper or lower gastrointestinal surgeons and all were male. One gastroenterologist was female. Videogame usage was low among all participants.

**Table 3.** Demographic Data.^a^

	Group A (Experts)	Group B (Novices)	Group C (Gastroenterologists)
n	4	7	3
Age, years	35.5 (33-52)	34 (31-36)	33 (33-46)
Postgraduate year of training (PGY)^b^	9 (6-30)	7 (4-12)	9 (2-20)
Male, %	100	100	66.6
Right-handed, %	75	100	100
Upper gastrointestinal surgeons	1	3	0
Lower gastrointestinal surgeons	1	3	0
Breast surgeons	0	1	0
Unspecialized	1	0	0
Gastroenterologists	1	0	3

a Continuous values quoted as median with range in parentheses.

b Only years with >50% clinical practice included.

**Table 4.** Procedures Performed Independently by Participants.^a^

	Group A (Experts)		Group B (Novices)		Group C (Gastroenterologists)	
	Animals or Simulators	Humans	Animals or Simulators	Humans	Animals or Simulators	Humans
Esophagoduodenogastroscopy	10 (0-25)	5 (0-80)	0 (0-55)	55 (5-150)	10 (5-55)	1000 (800-6000)
Small bowel enteroscopy	0 (0-0)	0 (0-0)	0 (0-0)	0 (0-0)	0 (0-5)	20 (0-200)
Colonoscopy	0.5 (0-20)	30 (0-20 000)	0 (0-100)	10 (0-50)	0 (5-20)	800 (250-3000)
Flexible sigmoidoscopy	5 (0-20)	30 (0-3000)	0 (0-5)	20 (0-50)	0 (0-0)	1000 (200-1000)
Any endoscopic procedure	18 (0-60)	105 (0-23 000)	10 (0-100)	75 (25-212)	30 (10-60)	2820 (1250-10 200)
Any laparoscopic procedure	12.5 (0-200)	80 (0-350)	20 (2-120)	120 (0-250)	0 (0-0)	0 (0-0)
Any natural orifice transluminal endoscopic surgery (NOTES)	12.5 (10-20)	1.5 (0-4)	0 (0-3)	0 (0-0)	0 (0-3)	0 (0-0)

a Continuous values quoted as median with range in parentheses.

### Face Validity

**Figure 5.**
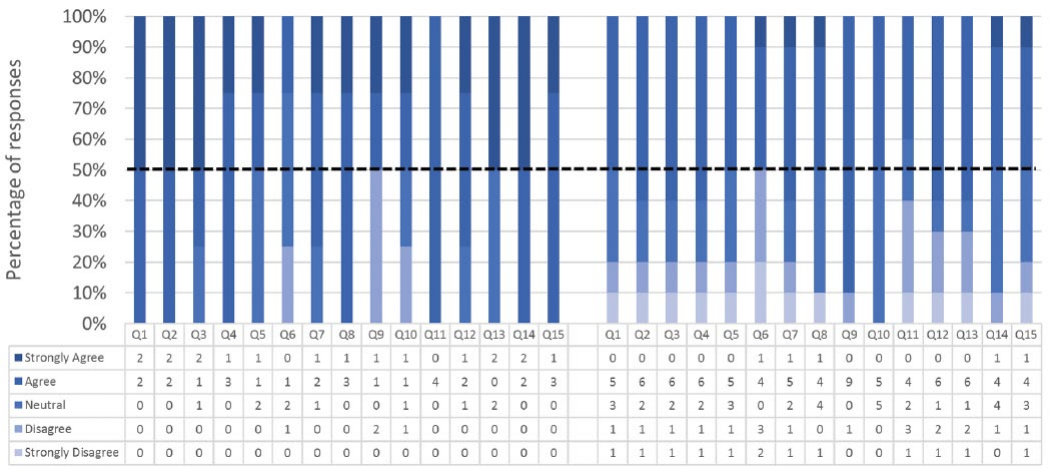
Face validity – Experts (on the left) and non-Experts (on the right) responses. Please refer to Table 2 for the survey questions.

Responses to the 15 statements pertaining to face validity are shown in Figure 5. In 14 out of 15 statements ≥50% of all participants’ responses were positive (ie, “agreed” or “strongly agreed”). A majority (n = 9) of participants indicated that the simulator, as well as the difficulty of the simulated procedure, were realistic. Participants felt that the amount and nature of looping of the endoscope was the most realistic aspect of the simulation and they were mainly dissatisfied with the feel of the endoscope hardware. In general, the Experts were more favorable in terms of face validity than Nonexperts. They fully “agreed” or “strongly agreed” with 7 statements and none of them “strongly disagreed” with any of the statements. Free text comments pertaining to face validity are shown in Table 5.

**Table 5.** Face Validity—Free Text Responses.

• “All or any comments I could make would be petty as it is a very nice simulator. Well done! Perhaps the weight of control handle and passive torque on shaft could be lightened.” (Expert) • “The instrument is much too stiff and heavy.” (Expert) • “Scope very clumsy to handle. Far too long and stiff; however, movement of tip was realistic. Felt like positioning on the gallbladder for dissection was more luck then judgement but I am not used to the anatomy.” (Gastroenterologist) • “Very unrealistic when compared to performing a laparoscopic cholecystectomy in a person.” (Novice) • “Insertion length was short. Sometimes you can push through a loop a bit.” (Novice)

### Content Validity

**Figure 6.**
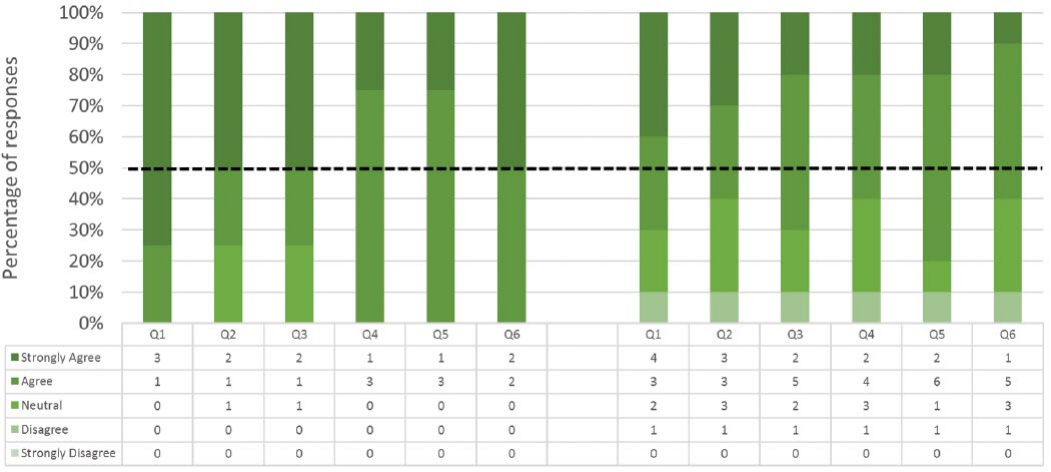
Content validity—Experts (on the left) and Nonexperts (on the right) responses. Please refer to Table 2 for the survey questions.

Responses to the 6 statements pertaining to content validity are shown in Figure 6. All Experts and 8 out of 10 Nonexperts agreed or strongly agreed that NOViSE can be a useful training tool for NOTES. All Experts and 6 out of 10 Nonexperts stated that they would recommend the simulator to others. Similarly to face validity, the Experts were more affirmative about the content validity than Nonexperts. Free text comments are shown in Table 6.

**Table 6.** Content Validity—Free Text Responses.

• “No current secure clip available for endoscope. Current transgastric cholecystectomy performed using fundal first technique then endoloops to CD.” (Expert) • “This simulator is still a work in progress so therefore is difficult to comment on whether or not this is a good training tool. As I have not performed NOTES surgery I cannot comment on its effectiveness as a training tool. Intuitively any simulator should help with real world surgery—but this is dependent on the fidelity and responsiveness of the simulator.” (Novice) • “Refinement required with clipping and cutting.” (Novice) • “Not sure if realistic or not as have never done a NOTES cholecystectomy, but feel scope needs to be easier to handle so very fine movements can be practiced.” (Gastroenterologist)

### Construct Validity

**Figure 7.**
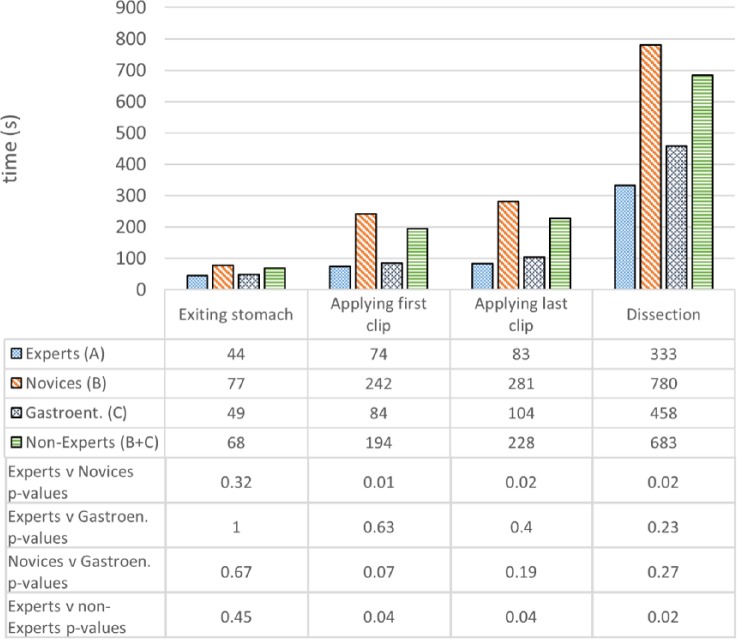
Construct validity—task completion times.

**Figure 8.**
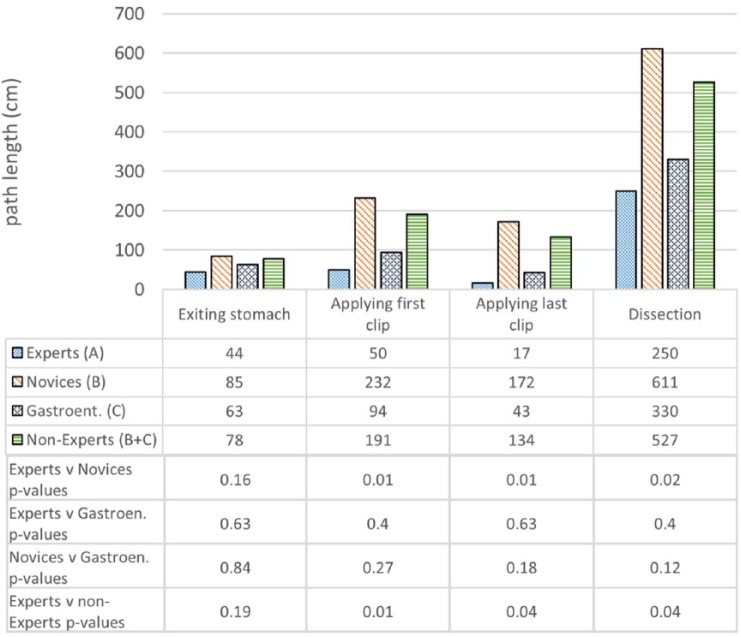
Construct validity—endoscope path lengths.

**Figure 9.**
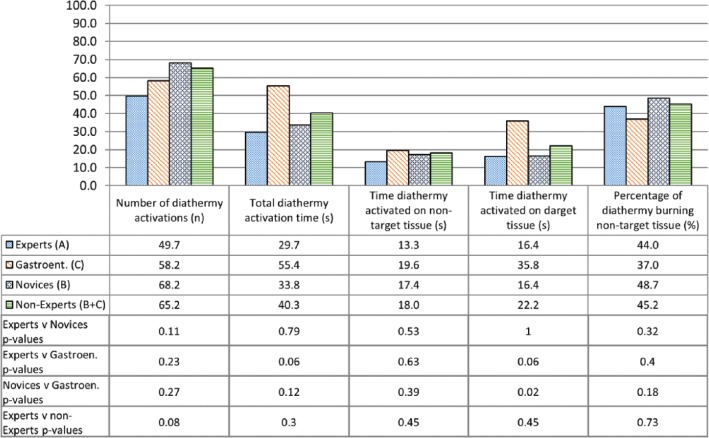
Construct validity—diathermy use.

Operative metrics and the corresponding *P* values are shown in Figures 7-9. Construct validity was demonstrated for the following simulator metrics between Experts and Novices (group A vs group B): time from exiting stomach to application of first clip (74 vs 242 seconds, *P* = .01), time from application of first clip to application of last clip (83 vs 281 seconds, *P* = .02), time from application of last clip to completed dissection of gallbladder from liver bed (333 vs 780 seconds, *P* = .02), endoscope path length from exiting stomach to application of first clip (50 vs 232 cm, *P* = .01), endoscope path length from application of first clip to dissection of cystic duct (17 vs 172 cm, *P* = .01), endoscope path length from dissection of cystic duct to complete dissection of gallbladder from liver bed (250 vs 611 cm, *P* = .02).

Construct validity was demonstrated for the same metrics between Experts and Nonexperts (group A vs group B + C): time from exiting stomach to application of first clip (74 vs 194 seconds, *P* = .04), time from application of first clip to application of last clip (83 vs 228 seconds, *P* = .04), time from application of last clip to completed dissection of gallbladder from liver bed (333 vs 683 seconds, *P* = .02), endoscope path length from exiting stomach to application of first clip (50 vs 191 cm, *P* = .01), endoscope path length from application of first clip to dissection of cystic duct (17 vs 134 cm, *P* = .04), endoscope path length from dissection of cystic duct to complete dissection of gallbladder from liver bed (250 vs 527 cm, *P* = .04).

Construct validity was not demonstrated for the remaining metrics between Experts and Novices or between Experts and Nonexperts. Construct validity was also not demonstrated for any metrics between Experts and Gastroenterologists, except for the time diathermy was activated on target tissue (16.4 vs 35.8 seconds, *P* = .02). Construct validity was not demonstrated for any metrics between Novices and Gastroenterologists.

## Discussion

This study has established the face, content, and construct validity of NOViSE—the first force-feedback enabled VR simulator for NOTES. NOViSE has shown good overall face and content validity, with main suggestions for improvement related to the feel of the haptic device and the design of the hand piece. Participants agreed that NOViSE is sufficiently realistic, that it can be a useful training tool for NOTES and that they would recommend it to others.

NOViSE also demonstrated early signs of construct validity. Experts were faster and used a shorter endoscopic path length than novices in all but the first task. The first task required participants to navigate from the esophagus, through a preexisting incision in the stomach and into the peritoneal cavity. The fact that no statistically significant intergroup difference was noted here is possibly because this task only requires relatively basic endoscopic navigation skills. The assisting investigators noted that many novices were aware of simple techniques such as aligning the distal endoscope adjacent to the gastric incision, then “tipping” it through with the controls. However, after entering the peritoneal cavity, participants were forced to navigate without the aid of a lumen supporting the shaft of the endoscope. Without this support, the shaft is more likely to form loops. This increases the complexity of the procedure as the endoscope does not behave in the anticipated manner: when the endoscope’s shaft is straight and the tip is in the neutral position, advancing the shaft will result in the tip of the scope moving directly ahead, making it easy to navigate toward visualized tissues. However, when the shaft of the endoscope is looped, even with the tip in the neutral position, advancing the shaft may result in the tip deviating to one side. Alternatively, the loop may prevent there being sufficient length of endoscope to reach the target tissue and advancing the shaft may even result in the tip moving away from it. The problem is compounded by the fact that the operator can rarely see that a loop has formed. The assisting investigator noted that novices found navigation with looped shafts very challenging; as was clearly reflected by their increased operative time and endoscope path length compared to the experts. Experts and novices did not differ in the forces that they applied to the tissues with the tip of the endoscope, endoscope shaft velocity or acceleration. This suggests that novices knew how to manipulate the endoscope safely, albeit not as efficiently as experts.

During cystic artery and duct clipping and cutting, experts were clearly more aware of how to position the endoscope tip to be able to efficiently clip and cut the designated points by steering only with the thumbwheels. Novices, on the other hand, often had to reposition the whole endoscope, which took considerably more time and longer paths. The assisting investigator noted that experts were more likely to ask for their help. Mainly, to hold the physical shaft while high torque was present. This enabled them to steer the tip more precisely and protrude/intrude the clipping tool using both hands. There was, however, no significant difference in terms of overall clipping and cutting accuracy. All groups had similar deviation from the optimal clipping and cutting point and angle, deployed similar number of clips and used similar instrument protrusion.

In NOTES, surgeons cannot manipulate tissues to expose anatomical planes while simultaneously using diathermy. The operator must first stabilize the endoscope, and then have an assistant advance the diathermy probe toward the target tissue. It is therefore perhaps surprising that no significant difference was found between experts and novices in terms of diathermy accuracy. This may be attributed to the fact that novices were aware of the need to avoid damage to healthy tissue (which they could identify given their anatomical and procedural knowledge) and able to activate the diathermy at appropriate times. Nevertheless, such a precision required a careful navigation and, as in the case of the other tasks, novices were significantly slower and travelled longer paths than experts during this task.

In relation to the relative performance of experts versus gastroenterologists and novices versus gastroenterologists, there was no significant difference in any of the metrics but one. However, a trend can be observed indicating that the gastroenterologists were faster and used shorter path lengths than novices in all tasks. This does correlate with the findings of Nehme et al,^13^ who found prior endoscopic experience to be of greater benefit than prior laparoscopic experience in acquiring skills in NOTES.

The main limitations of NOViSE are that it currently only simulates transgastric hybrid cholecystectomy and, owing to the fact that it is a prototype, some features of the hardware, such as the endoscope’s length and visual realism, as well as the use of foot pedals to activate the clipper and scissors, can be refined for improved performance. The same is true of certain aspects of the simulation software, such as the absence of fat in Calot’s triangle.

Our study suffered from small numbers in each of the participant groups as there is only 1 institution that performs NOTES so far in the United Kingdom. There was also a large variation in procedural experience within each group. Participants completed their procedures at times convenient to them, which meant that some participants performed all three procedures in sequence, whilst others performed single procedures separated by several days or weeks. This may have influenced the results depending on whether repeated operating led to fatigue or greater familiarity with the procedure. The statements for face and construct validity were phrased so that agreement correlated with validity. This may have introduced acquiescence bias. Future studies should address this by using a balance of positively and negatively phrased questions.

Future work includes upgrading the simulator software and hardware to address the identified shortcomings. We also plan to support a wider range of NOTES procedures (eg, appendectomy) and approaches (transvaginal, possibly transrectal), as well as advanced endoscopy such as endoscopic mucosal resection and endoscopic submucosal dissection. Features such as dissection of Calot’s triangle, creation and closure of viscerotomy will also be added. After implementing these improvements, further validation studies on a larger group of participants will be conducted.
